# Formal axioms in biomedical ontologies improve analysis and interpretation of associated data

**DOI:** 10.1093/bioinformatics/btz920

**Published:** 2019-12-10

**Authors:** Fatima Zohra Smaili, Xin Gao, Robert Hoehndorf

**Affiliations:** Computer, Electrical & Mathematical Sciences and Engineering (CEMSE) Division, Computational Bioscience Research Center (CBRC), King Abdullah University of Science and Technology (KAUST), Thuwal, Saudi Arabia

## Abstract

**Motivation:**

Over the past years, significant resources have been invested into formalizing biomedical ontologies. Formal axioms in ontologies have been developed and used to detect and ensure ontology consistency, find unsatisfiable classes, improve interoperability, guide ontology extension through the application of axiom-based design patterns and encode domain background knowledge. The domain knowledge of biomedical ontologies may have also the potential to provide background knowledge for machine learning and predictive modelling.

**Results:**

We use ontology-based machine learning methods to evaluate the contribution of formal axioms and ontology meta-data to the prediction of protein–protein interactions and gene–disease associations. We find that the background knowledge provided by the Gene Ontology and other ontologies significantly improves the performance of ontology-based prediction models through provision of domain-specific background knowledge. Furthermore, we find that the labels, synonyms and definitions in ontologies can also provide background knowledge that may be exploited for prediction. The axioms and meta-data of different ontologies contribute to improving data analysis in a context-specific manner. Our results have implications on the further development of formal knowledge bases and ontologies in the life sciences, in particular as machine learning methods are more frequently being applied. Our findings motivate the need for further development, and the systematic, application-driven evaluation and improvement, of formal axioms in ontologies.

**Availability and implementation:**

https://github.com/bio-ontology-research-group/tsoe.

**Supplementary information:**

Supplementary data are available at *Bioinformatics* online.

## 1 Introduction

Biomedical ontologies are widely used to formally represent the classes and relations within a domain and to provide a structured, controlled vocabulary for the annotations of biological entities (Smith *et al.*, 2007). Over the past years, significant efforts have been made to enrich ontologies by incorporating formalized background knowledge as well as meta-data that improve accessibility and utility of the ontologies (Mungall *et al.*, 2011; Smith *et al.*, 2007). Incorporation of formal axioms contributes to detecting whether ontologies are consistent (Smith and Brochhausen, 2010; Smith *et al.*, 2003; Stevens *et al.*, 2003), enables automated reasoning and expressive queries (da Silva *et al.*, 2017; Hoehndorf *et al.*, 2015a; Jupp *et al.*, 2012), facilitates connecting and integrating ontologies of different domains through the application of ontology design patterns (Hoehndorf *et al.*, 2010; Osumi-Sutherland *et al.*, 2017) and can be used to guide ontology development (Alghamdi *et al.*, 2019; Köhler *et al.*, 2013).

While axioms are mainly exploited through automated tools and methods, ontologies also contain labels, synonyms and definitions (Hoehndorf *et al.*, 2015b); improving the human-accessible components of ontologies has also been a major focus of ontology development (Köhler *et al.*, 2006); for example, including ‘good’ natural language definitions and adequate labels is a requirement for biomedical ontologies in the Open Biomedical Ontologies Foundry (Smith *et al.*, 2007), an initiative to collaboratively develop a set of reference ontologies in the biomedical domains.

The amount of information contained in ontologies, and the rigour with which this information has been created, verified and represented, may also improve domain-specific data analysis through the provision of background knowledge (Garcez and Lamb, 2004). Domain-specific background knowledge can limit the solution space in optimization and search problems (Besold *et al.*, 2017; Garcez and Lamb, 2004; Garcez *et al.*, 2015) and therefore allow finding solutions faster or finding better, more generalized solutions. Ontologies and formalized biological knowledge could therefore crucially improve machine learning and applications of Artificial Intelligence in biology.

The Gene Ontology (GO) (Ashburner *et al.*, 2000) is a biomedical ontology that formally represents several aspects of biological systems, in particular the molecular functions that gene products may have, the biological processes they may be involved in, and the cellular components in which they are located (Huntley *et al.*, 2014b). The GO has been extensively used to provide annotations to gene products through a combination of manual curation of literature and electronic assignments created using algorithms based on sequence similarity, keywords, domain information and others (Huntley *et al.*, 2014a). Databases such as the GO annotation (GOA) database (Huntley *et al.*, 2015) use GO to annotate more than 50 million proteins.

Due to its central role and importance in molecular biology, significant resources have been invested in the development of GO. Over the years, substantial efforts have been made to improve the coverage of GO through the addition of new classes (Consortium, 2014, 2016). In addition to new classes, GO has also been extended through axioms that characterize the intended meaning of a class formally (Mungall *et al.*, 2011). Specifically, GO now includes links between GO classes and classes in other biomedical ontologies (Bada and Hunter, 2008) in an extended version of GO (which we refer to as ‘GO-Plus’) (Consortium, 2014, 2016). These axioms are particularly useful in keeping GO complete and logically consistent with other ontologies as well as in guiding ontology development (Bodenreider and Burgun, 2005; Consortium, 2016; Johnson *et al.*, 2006; Mungall *et al.*, 2011). There are now more than 90 000 inter-ontology axioms in GO-Plus that weave GO together with several other ontologies in the biomedical domain.

While these axioms have primarily been developed to tackle the problem of continuously developing GO while maintaining consistency (within GO and other ontologies) as well as to maintain biological accuracy, they have also the potential to significantly improve GO-based data analysis by introducing new associations between classes that are not present in GO but arise through information in other, related ontologies. For example, the GO classes *Histidine catabolic process to glutamate and formamide* (GO: 0019556) and *Formamide metabolic process* (GO: 0043606) are not directly (or closely) related in the GO hierarchy but both are related to the ChEBI (Chemical Entities of Biological Interest) class *Formamide* (ChEBI: 16397) through axioms formulated in the Web Ontology Language (OWL) (Grau *et al.*, 2008), a formal language based on Description Logics (Horrocks *et al.*, 2006). If a data analysis method can utilize the axioms in this formal language, we expect improved performance results when applied to different domains.

A task or method that explicitly relies on the axioms or the meta-data in ontologies can be used not only to improve data analysis but also to evaluate the ‘quality’ of axioms in ontologies in contributing to such an analysis task (Hoehndorf *et al.*, 2013). Specifically, such a method would enable determining whether axioms and formalized knowledge contribute to biomedical data analysis, and allow evaluating and comparing how much they contribute to particular tasks.

Recently, several machine learning methods became available that make it possible to utilize different components of ontologies—axioms, labels, definitions and other kinds of meta-data—in machine learning tasks without the need for manual extraction of features (which may introduce a bias). Here, we use recently developed techniques, Onto2Vec (Smaili *et al.*, 2018a), OPA2Vec (Smaili *et al.*, 2018b) and Node2Vec (Grover and Leskovec, 2016), to predict protein interactions based on functional information and gene–disease associations based on phenotypes. We evaluate the effect of the axioms that have been added to the GO as well as the effect of adding the axioms of additional domain ontologies as the background knowledge. We demonstrate that the formal axioms that have been created for GO and other ontologies improve predictive data analysis by providing background knowledge about biological domains. Our approach is also applicable to evaluation of meta-data such as labels and definitions and their contribution to predictive analysis of biomedical data. We find that labels and definitions in ontologies can fill gaps in domain knowledge that are not covered by the axioms and further improve prediction; however, the labels and definitions have also the potential to add noise or bias to prediction results. Finally, through our analysis we also improve the performance of predicting protein interactions and gene–disease associations through ontologies. Overall, our results demonstrate the value that ontologies can provide to biomedical data analysis not merely through their provision of controlled vocabularies but also because they are richly formalized knowledge bases and sources of definitions of domain entities.

## 2 Materials and methods 

### 2.1 Ontologies

#### 2.1.1 GO and GO-Plus

We downloaded the GO (Ashburner *et al.*, 2000) in OWL (Grau *et al.*, 2008) format from http://www.geneontology.org/ontology/ on April 14, 2018. This version of GO contains 107 762 logical axioms. We also downloaded the GO protein annotations from the UniProt-GOA website (http://www.ebi.ac.uk/GOA) on December 2, 2018. All associations with evidence code IEA were filtered, which results in a total of 3 474 539 associations for 749 938 unique proteins.

GO-Plus (downloaded from http://purl.obolibrary.org/obo/go/extensions/go-plus.owl) is an extension of GO that contains, in addition to all the logical axioms of GO, additional inter-ontology axioms that describe relationships between GO classes and other external biomedical ontologies, in particular: ChEBI ontology (Degtyarenko *et al.*, 2007), PO (The Plant Ontology) (Jaiswal *et al.*, 2005), CL (The Cell Ontology) (Bard *et al.*, 2005), PATO (Phenotype and Trait Ontology) (Gkoutos *et al.*, 2004, 2017), the Uberon ontology (Mungall *et al.*, 2012), SO (The Sequence Ontology) (Eilbeck *et al.*, 2005), FAO (Fungal gross anatomy), OBA (Ontology of Biological Attributes), NCBITaxon (NCBI organismal classification), CARO (Common Anatomy Reference Ontology) (Haendel *et al.*, 2008) and PR (Protein Ontology) (Natale *et al.*, 2011). A detailed description of each one of these ontologies is provided in Supplementary Material. In addition, Supplementary Table S2 summarizes the number of axioms in GO-Plus describing relations to each of these ontologies and shows an example of such axioms for each ontology.

#### 2.1.2 PhenomeNET ontology

We downloaded the PhenomeNET ontology (Hoehndorf *et al.*, 2011; Rodríguez-García *et al.*, 2017) in OWL format from the AberOWL repository http://aber-owl.net (Hoehndorf *et al.*, 2015a) on February 21, 2018. PhenomeNET is a cross-species phenotype ontology that combines phenotype ontologies, anatomy ontologies, GO and several other ontologies in a formal manner (Hoehndorf *et al.*, 2011).

### 2.2 Evaluation datasets

#### 2.2.1 Protein–protein interactions

To evaluate our work, we predict protein–protein interaction (PPI) on three different organisms: human, yeast and *Arabidopsis thaliana*. The datasets for all three organisms were obtained from the STRING database (Szklarczyk *et al.*, 2017). The human dataset contains 19 577 proteins and 11 353 057 interactions, the yeast dataset contains 6392 proteins and 2 007 135 interactions, while the *Arabidopsis* dataset contains 10 282 070 interactions for 13 261 proteins.

#### 2.2.2 Gene–disease associations

To further evaluate our method, we predict gene–disease associations. The first dataset used in this experiment is the mouse phenotype annotations obtained from the Mouse Genome Informatics (MGI) database (Smith and Eppig, 2015) on February 21, 2018 with a total of 302 013 unique mouse phenotype annotations. The second dataset used for this experiment is the disease to human phenotype annotations obtained on February 21, 2018 from the Human Phenotype Ontology (HPO) database (Robinson *et al.*, 2008). We limited our analysis to the OMIM diseases only which resulted in a total of 78 208 unique disease-phenotype associations. To validate our prediction, we used the MGI_DO.rpt file from the MGI database to obtain 9506 mouse gene–OMIM disease associations and 13 854 human gene–OMIM disease associations. To map mouse genes to human genes we used the HMD_HumanPhenotype.rpt file from the MGI database.

### 2.3 Analysis algorithms

Our analysis is based on prediction results obtained using embeddings of biological entities (proteins, genes, diseases) obtained from ontologies using two tools: Onto2Vec (Smaili *et al.*, 2018a) and OPA2Vec (Smaili *et al.*, 2018b). The obtained embeddings are then trained using a neural network (NN) to make predictions.

#### 2.3.1 Onto2Vec

Onto2Vec (Smaili *et al.*, 2018a) is a method that uses ontologies to obtain embeddings of ontology classes and the entities they annotate. Onto2Vec uses two main information sources: First, it used all logical axioms describing the structure of an ontology including both asserted axioms of an ontology as well as inferred axioms using a semantic reasoner. Second, it uses the known ontology-based associations of biological entities (e.g. protein–GO associations). These two pieces of information form a corpus of text used to train Word2Vec (Mikolov *et al.*, 2013a, b) and obtain the embeddings.

#### 2.3.2 OPA2Vec

OPA2Vec (Smaili *et al.*, 2018b) is also a tool used to obtain embeddings of biological entities from ontologies. In addition to using logical axioms, OPA2Vec also uses annotation property axioms from the ontology meta-data. These annotation axioms use natural language to describe different properties of the ontology classes (labels, descriptions, synonyms, etc.) and they, therefore, form a rich corpus of text for Word2Vec. To provide the Word2Vec model with some background knowledge on the ontology concepts described by the annotation properties, OPA2Vec pre-trains the model on a corpus of biomedical text (PubMed by default). Entity-class annotations are also used an additional source of information to produce the ontology-based embeddings of biological entities.

#### 2.3.3 Node2Vec

We use Node2Vec (Grover and Leskovec, 2016) to obtain entity embeddings from the biomedical ontologies and their annotations. Node2Vec is a model that learns embeddings of nodes in a graph by applying the Word2Vec model on sequences of nodes. Here, we apply Node2Vec on the ontology graph consisting of subclass, equivalence and disjointness axioms as well as all types of axioms involving exactly two classes. We use relational patterns (Hoehndorf *et al.*, 2010) to map some axioms to graph edges: we identify all axioms of the type *X* SubClassOf: *R* some *Y* and *X*EquivalentTo: *R* some *Y* (with *X*, *Y* and *R* being variables) and for each triple (*X*, *Y*, *R*) we assign an edge labelled *R* from *X* to *Y* (bidirectional in the case of EquivalentTo:). In addition to these relations, we also represent the annotated entities as a direct edge. The detailed parameters used to execute the Node2Vec algorithm are shown in Supplementary Table S2 and the general workflow of how we used Node2Vec is shown in Supplementary Figure S3.

### 2.4 Neural network training and optimization

To optimize our prediction models (PPI and gene–disease associations predictions), we train a NN using the obtained embeddings from Onto2Vec, OPA2Vec and Node2Vec. Limited grid search has been performed to select a suitable NN for our predictions based on suggested guidelines (Hunter *et al.*, 2012). The chosen NN is a feed-forward network with two hidden layers of 800 and 200 neurons, respectively. The NN is optimized using binary cross entropy as the loss function. We train our model on 70% of our data and test on the remaining 30% without performing cross-validation.

## 3 Results

### 3.1 Contribution of axioms in PPI prediction

We follow a strategy for the external evaluation of ontologies (Hoehndorf *et al.*, 2013) and apply the method to the task of predicting interactions between proteins and gene–disease associations. Specifically, we test the impact of ontology axioms and ontology meta-data on machine learning applications that rely on ontologies. For this purpose, we use a basic version of the GO (Ashburner *et al.*, 2000) as the baseline, implement an ontology-based prediction workflow and evaluate the results. We then compare the performance of ontology-based predictive analysis to the use of GO-Plus in the same workflow and evaluate the results on the same evaluation set. GO-Plus is GO with a large set of formal axioms added that define and constrain GO classes and connect them to classes that are defined in other ontologies (Mungall *et al.*, 2011). Furthermore, we add additional background knowledge of the form of the complete set of axioms in biomedical ontologies that are explicitly used in the GO-Plus axioms, and evaluate their impact on predictive performance. Throughout these experiments, we keep training and testing data (comprised protein identifiers with their associated GO classes) fixed, changing only the amount of background information available through GO.

Since GO-Plus combines all axioms existing in GO with additional axioms that describe relations to other biomedical ontologies, we expect GO-Plus in combination with the axioms and the meta-data of other ontologies to improve predictive performance. We first apply GO and GO-Plus to the task of predicting PPIs, and to account for possible differences between taxa in predicting PPIs, we evaluate our hypothesis on human, yeast and *Arabidopsis* proteins and their interactions.

To predict PPIs using GO and GO-Plus, we assign GO functions to human, yeast and *Arabidopsis* proteins based on their annotations in the GOA database (Huntley *et al.*, 2015). We then apply the Onto2Vec method (Smaili *et al.*, 2018a), using either GO or GO-Plus as background knowledge, to obtain ontology embeddings of the proteins. An ontology embedding is a function that maps entities from an ontology (and its annotations) into an *n*-dimensional vector space (Smaili *et al.*, 2018b), and Onto2Vec encodes for ontology-based annotations of entities together with all the axioms in the ontology (Smaili *et al.*, 2018a). We further use the Node2Vec (Grover and Leskovec, 2016) method on the graph structure generated from GO and GO-Plus to generate embeddings (details on how we use Node2Vec to generate the embeddings can be found in Section 2.3.3).

Our workflow generates features (embeddings) for proteins based on the same set of GOAs but utilizes different sets of axioms (either the axioms in GO, or the extended set of axioms in GO-Plus), and therefore allows us to evaluate the contribution of the ontology axioms to predictions based on these features.

We use the generated features to predict PPIs in two different ways: first, we calculate the cosine similarity between pairs of protein feature vectors (generated through Onto2Vec/Node2Vec), and second, we train a four-layer fully connected NN on pairs of vectors (see Section 2.4), and use a sigmoid output to obtain a prediction confidence score (Onto2Vec-NN/Node2Vec-NN). We evaluate the results of both prediction methods.

**Fig. 1.**
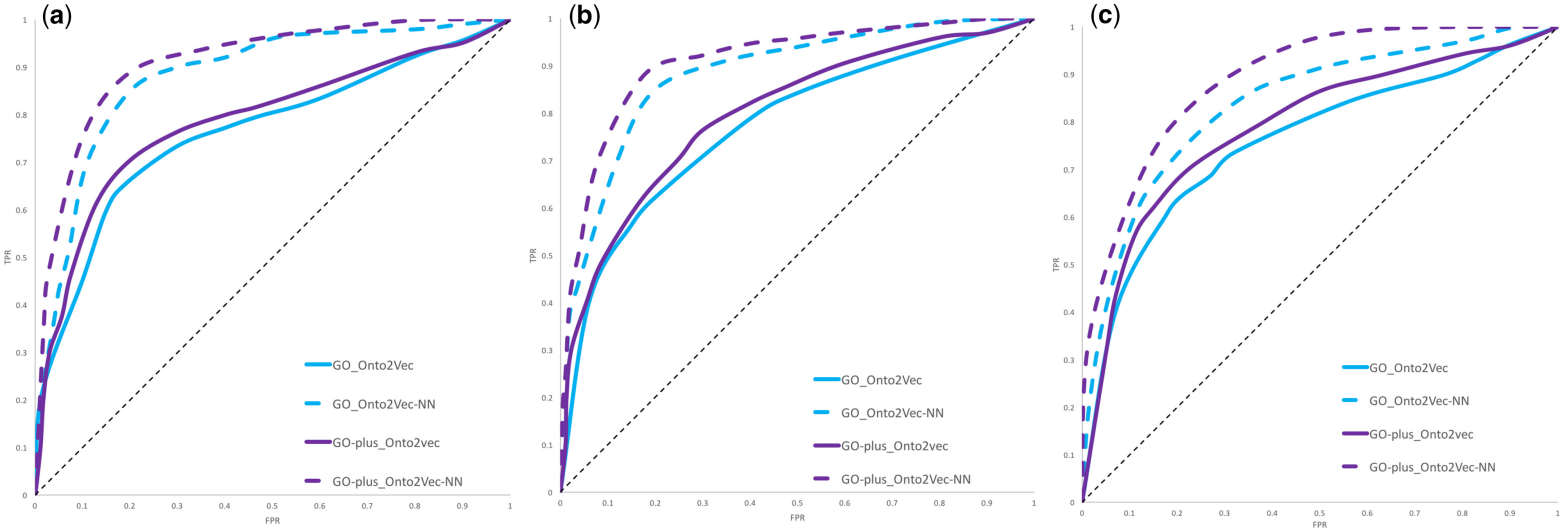
ROC curves for PPI prediction using GO and GO-Plus based on Onto2Vec and Onto2Vec-NN for (**a**) human, (**b**) yeast and (**c**) *A. thaliana*

Table 1 shows the corresponding AUC values for PPI prediction on GO and GO-Plus using Onto2Vec and Node2Vec. The ROC curves for PPI prediction for GO and GO-Plus using both Onto2Vec (cosine similarity) and Onto2Vec-NN (NN) for human, yeast and *A. thaliana* are shown in Figure 1. The ROC curves obtained from using Node2Vec for PPI prediction care shown in Supplementary Figure S1.

**Table 1.** AUC values of ROC curves for PPI prediction for GO-Plus and GO using Onto2Vec (cosine similarity) and Onto2Vec-NN (neural network) as well as using Node2Vec (cosine similarity) and Node2Vec_NN (neural network)

	Human	Yeast	*Arabidopsis*
GO_Onto2Vec	0.7660	0.7701	0.7559
GO_Onto2Vec_NN	0.8779	0.8711	0.8364
GO_plus_Onto2Vec	0.7880	0.7943	0.7889
GO_plus_Onto2Vec_NN	**0.9021**	**0.8937**	**0.8834**
GO_Node2Vec	0.7648	0.7671	0.7601
GO_Node2Vec_NN	0.8431	0.8568	0.8245
GO_plus_Node2Vec	0.7713	0.7802	0.7751
GO_plus_Node2Vec_NN	0.8794	0.8762	0.8573

*Note*: The best AUC value for each data set is shown in bold.

Our results show that the PPI prediction performance obtained from feature vectors generated using GO-Plus (and the rich set of axioms it contains) outperforms the predictions obtained from using GO axioms alone, in both the unsupervised model (Onto2Vec) and the supervised model (Onto2Vec-NN). The improvement in predictive performance is significant for the Onto2Vec prediction based on cosine similarity (*P *=* *0.021 for human, *P *=* *0.034 for yeast, *P *=* *0.027 for *Arabidopsis*; Mann–Whitney *U*-test), and significant for human and *Arabidopsis* in the NN based models (*P *=* *0.047 for human, *P *=* *0.061 for yeast, *P *=* *0.039 for *Arabidopsis*; Mann–Whitney *U*-test). However, the improvement in performance using Node2Vec is significant for *Arabidopsis* using cosine similarity (*P *=* *0.063 for human, *P *=* *0.071 for yeast, *P *=* *0.044 for *Arabidopsis*; Mann–Whitney *U*-test) and for human and *Arabidopsis* using the NN (*P *=* *0.038 for human, *P *=* *0.060 for yeast, *P *=* *0.042 for *Arabidopsis*; Mann–Whitney *U*-test).

GO-Plus uses axioms from many biomedical ontologies but only includes small parts of these ontologies; we hypothesize that the axioms in the ontologies that are referenced in GO-Plus can contribute additional background knowledge that may further improve data analysis. Therefore, we evaluate the individual contribution of each of the ontologies used in GO-Plus axioms, i.e. we individually evaluate the axioms in the ChEBI ontology (Degtyarenko *et al.*, 2007), the PO (Jaiswal *et al.*, 2005), the Cell type Ontology (CL) (Bard *et al.*, 2005), the PATO (Gkoutos *et al.*, 2004, 2017), the Uberon ontology (Mungall *et al.*, 2012), the SO (Eilbeck *et al.*, 2005), the FAO, the OBA, the NCBITaxon, the CARO (Haendel *et al.*, 2008) and the PR (Natale *et al.*, 2011) (a detailed description of each ontology can be found in Section 2.1). We perform this evaluation using Onto2Vec only due to its ability to exploit different types of ontology axioms.

We repeat the same workflow as before to generate features: representation of GOAs of the proteins in human, yeast and *Arabidopsis*, and representation learning with Onto2Vec using GO-Plus as background knowledge; in each experiment we limit the axioms in GO-Plus to those that contain a reference to a particular ontology. We then again apply Onto2Vec to generate features and predict PPIs through cosine similarity or using a NN (Onto2Vec-NN) on human, yeast and *Arabidopsis*. The AUC values for the PPI prediction using GO-Plus but limited to the axioms that refer to a particular ontology are shown in Table 2. We observe that most of the inter-ontology axioms generally improve the predictive performance, with ChEBI contributing the most to improving PPI prediction and PATO improving the least (even decreasing the performance in some cases). The PO is a plant-specific domain ontology and improves predictive performance mainly when predicting PPIs in *Arabidopsis*, as can be expected.

**Table 2.** AUC values of the ROC curves for PPI prediction showing the contribution of the GO-Plus axioms corresponding to each ontology for human, yeast and *A. thaliana*

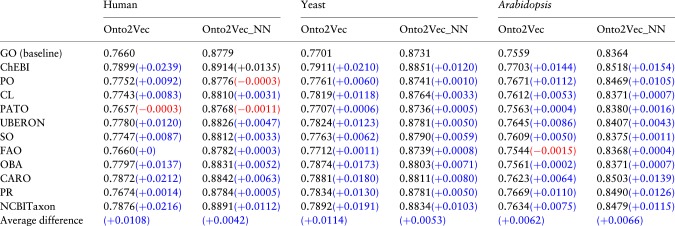

*Note*: The improvement (blue)/decrease (red) in performance of each ontology compared to GO is shown between parentheses. The last row shows the average difference of the performance across all ontologies compared to the GO baseline. (Color version of this table is available at *Bioinformatics* online.)

As a further experiment, we combine all ontologies, i.e. we add the complete set of axioms from each referenced ontology to the axioms of GO-Plus so that the background knowledge of the referenced ontology becomes available to Onto2Vec as well, and then apply our feature learning and prediction workflow. The detailed AUC values for predicting PPIs based on this comprehensive set of ontologies are shown in Supplementary Table S1. We observe a similar performance to using only the ontology-specific axioms in GO-Plus.

As a final experiment, we replace Onto2Vec with OPA2Vec to evaluate the contribution of ontology meta-data such as labels, synonyms and definitions, to their predictive performance. We again add each ontology that is referenced in a GO-Plus axiom to the axioms of GO-Plus, this time also including the meta-data (in the form of annotation axioms) of GO-Plus and the referenced ontology. OPA2Vec (pre-trained on the PubMed corpus) can encode both the axioms and meta-data of ontologies and observing the difference from the performance of Onto2Vec can therefore help to evaluate if—and how much—the labels, definitions and other meta-data contribute.

We again predict PPIs in two different ways: calculating the cosine similarity between the obtained protein feature vectors (referred to as OPA2Vec in the results table) and using the feature vectors to train a NN for PPI prediction (referred to as OPA2Vec-NN in the results tables). The obtained AUC values from this experiment compared to using GO are shown in Table 3. We find that the additional meta-data does, in general, not improve predictive performance; on the contrary, the predictive performance drops markedly when adding the meta-data in several ontologies, most notably PATO and ChEBI.

**Table 3.** AUC values of the ROC curves for PPI prediction for different external ontologies in GO-Plus using OPA2Vec and OPA2Vec-NN

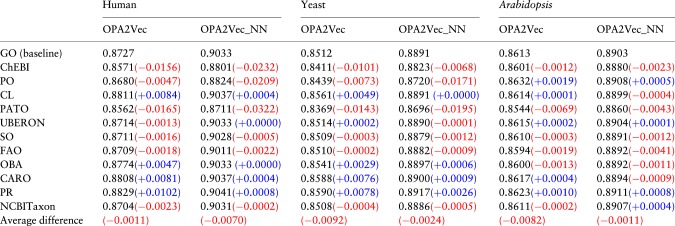

*Note*: Each prediction method uses the meta-data encoded in GO as well as the meta-data from the external ontologies. In each model, all logical axioms and annotation properties from GO, all logical axioms and all annotation properties from the external ontology and all GO-Plus inter-ontology axioms are included. The improvement (blue)/decrease (red) in performance of each ontology compared to GO is shown between parentheses. The last row shows the average difference of the performance across all ontologies compared to the GO baseline. (Color version of this table is available at *Bioinformatics* online.)

### 3.2 Gene–disease association prediction using GO-Plus

In our initial analysis we apply GO and GO-Plus to the task of predicting PPIs. Although we utilize PPI datasets from different species for the evaluation to generalize our results, it is nevertheless limited to prediction of PPIs and it is unclear if our results also hold for other types of predictive analysis.

We extend our analysis to the evaluation of predicting gene–disease associations based on phenotype similarity (Hoehndorf *et al.*, 2011). While GO is not a phenotype ontology, it is used in the axioms that make up most phenotype ontologies (Gkoutos *et al.*, 2017). We use the cross-species phenotype ontology PhenomeNET (Hoehndorf *et al.*, 2011; Rodríguez-García *et al.*, 2017), which relies on the GO for defining phenotypes, and replace the GO in PhenomeNET with GO-Plus.

**Fig. 2.**
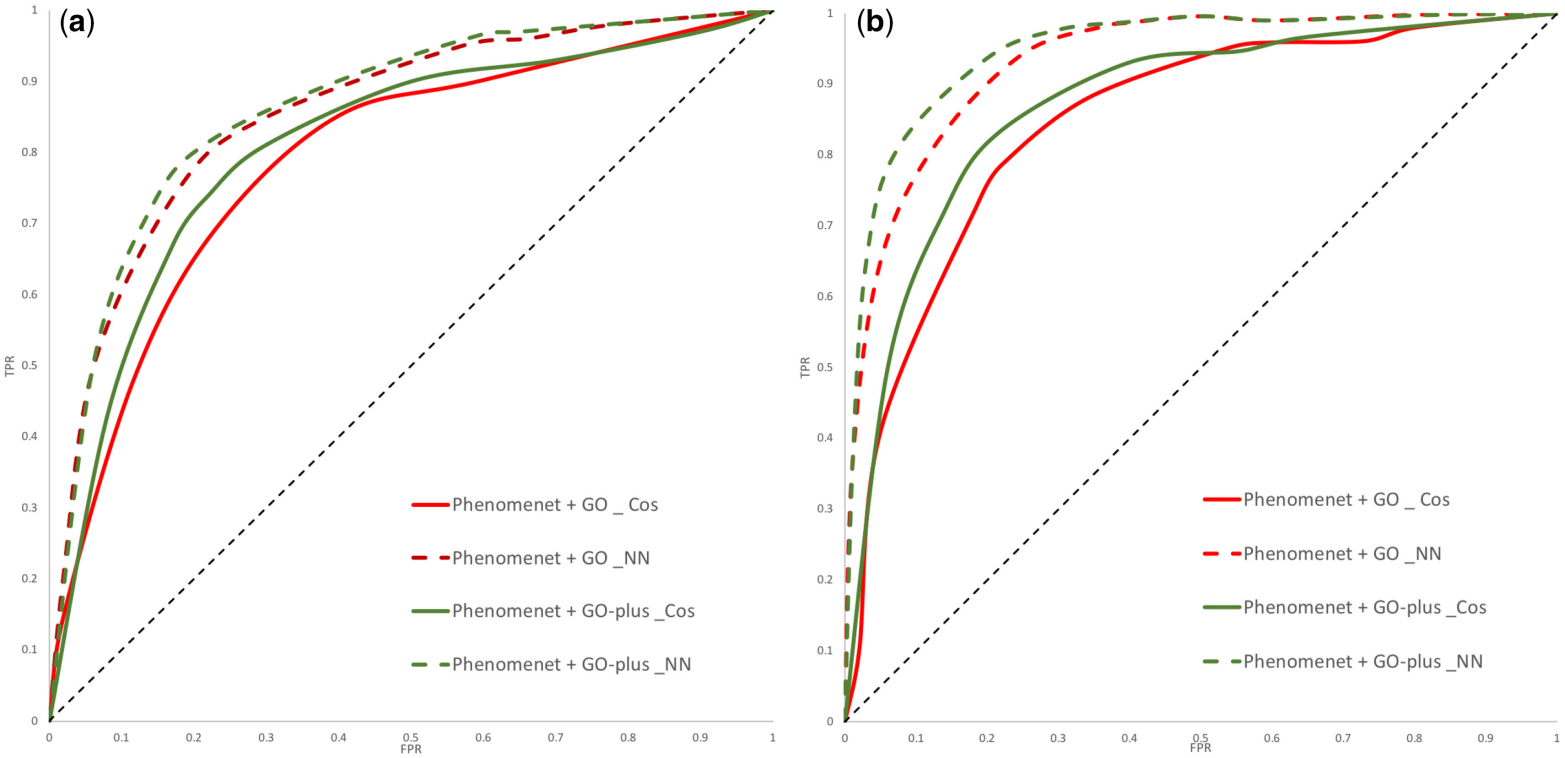
ROC curves for gene–disease prediction comparing PhenomeNET with GO (PhenomeNET + GO) to PhenomeNET with GO-Plus (PhenomeNET + GO-Plus) using Onto2Vec with cosine similarity (Cos) and with a NN for human gene–disease associations and mouse models of human disease. (**a**) Human and (**b**) mouse

**Fig. 3.**
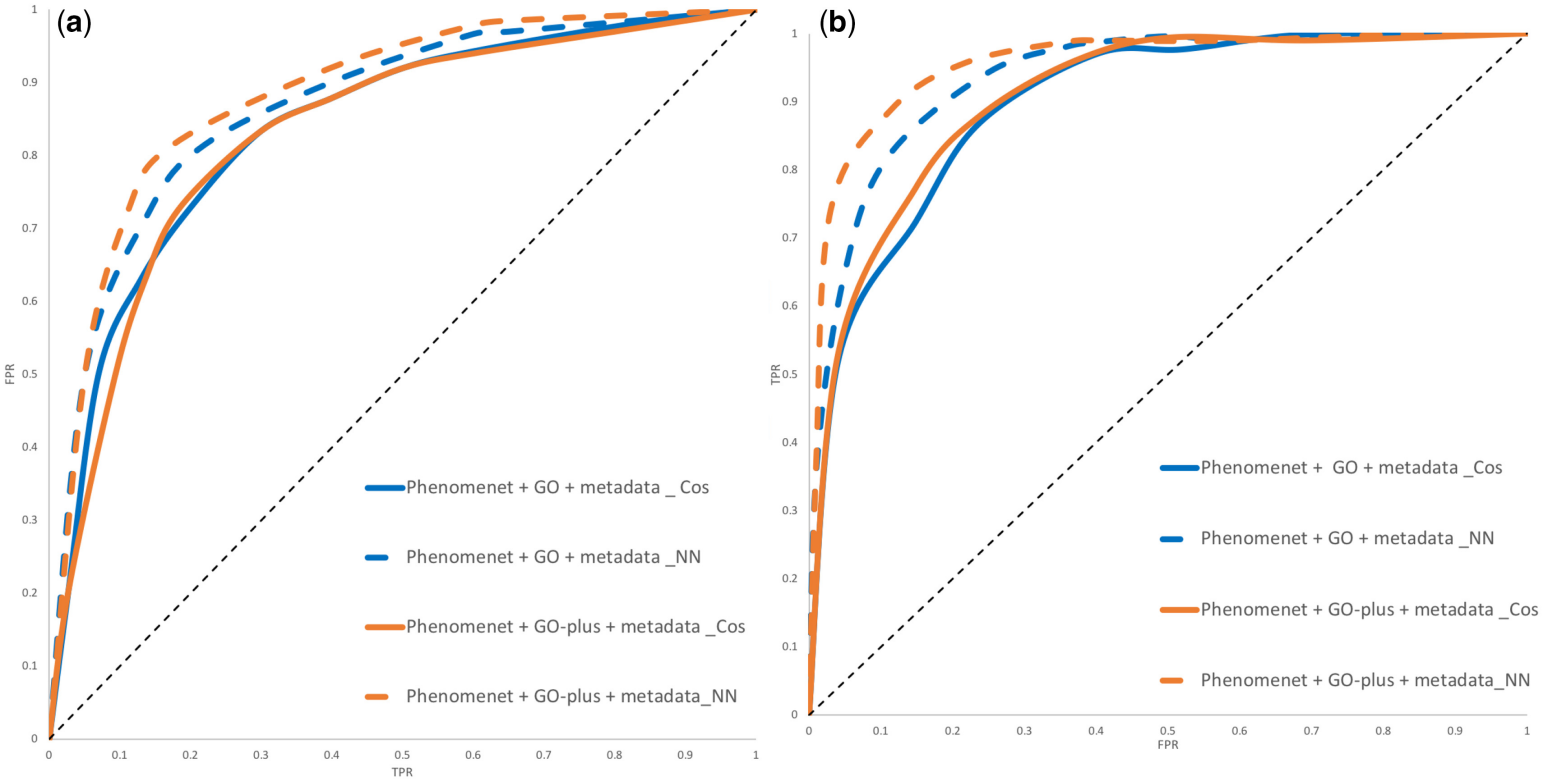
ROC curves for gene–disease prediction comparing PhenomeNET with GO with the metadata (PhenomeNET + GO + metadata) to PhenomeNET with GO-Plus (PhenomeNET + GO-Plus + metadata) using OPA2Vec with cosine similarity (Cos) and with a NN for human gene–disease associations and mouse models of human disease. (**a**) Human and (**b**) mouse

We annotate genes with mouse phenotypes from the MGI (Blake *et al.*, 2017) database as well as disease phenotypes from the HPO (Köhler *et al.*, 2017) database, and apply Onto2Vec, Onto2Vec-NN (Smaili *et al.*, 2018a) and Node2Vec (Grover and Leskovec, 2016) to encode these phenotypes and the axioms in PhenomeNET as feature vectors (more details on the gene–phenotype and disease–phenotype datasets can be found in Section 2.2). We then predict gene–disease associations or mouse models of human disease based on either cosine similarity or a NN using Onto2Vec, OPA2Vec and Node2Vec. We report the results in Table 4. The detailed ROC curve figures are shown in Figures 2 and 3. The ROC curves showing the Node2Vec-based results for gene–disease association prediction are reported in Supplementary Figure S2. The results show that the additional information that GO-Plus provides can significantly improve the overall prediction performance of PhenomeNET in predicting human gene–disease associations and mouse models of human disease (*P *=* *0.0411 for mouse and *P *=* *0.0254 for human, OPA2Vec, Mann–Whitney *U*-test).

**Table 4.** AUC values of ROC curves for gene–disease prediction using PhenomeNET and when replacing GO in PhenomeNET with GO-Plus as well as using Node2Vec with PhenomeNET and when replacing GO in PhenomeNET with GO-Plus

	Human	Mouse
Phenomenet+GO_Cos	0.7841	0.8431
Phenomenet+GO_NN	0.8461	0.9141
Phenomenet+GO-plus_Cos	0.7990	0.8507
Phenomenet+GO-plus_NN	0.8532	0.9182
Phenomenet+GO+metadata_Cos	0.8304	0.8651
Phenomenet+GO+metadata_NN	0.8595	0.9188
Phenomenet+GO-plus+metadata_Cos	0.8313	0.8672
Phenomenet+GO-plus+metadata_NN	**0.8761**	**0.9204**
Phenomenet+GO_Node2Vec	0.7604	0.8104
Phenomenet+GO_Node2Vec_NN	0.8003	0.8601
Phenomenet+GO_plus_Node2Vec	0.7794	0.8376
Phenomenet+GO_plus_Node2Vec_NN	0.8283	0.8882

*Note*: The best AUC value for each data set is shown in bold.

## 4 Discussion

We developed a method to evaluate the contribution of ontology axioms to computational analysis of biomedical data. We use feature learning methods which are generic and data-driven, and encode for a large set of information contained in ontologies. Our choice is motivated by the desire to avoid potential biases, and the ability to use a wide range of formal as well as informal information contained in biomedical ontologies. However, our evaluation is naturally limited to the choice of the embedding methods (Onto2Vec, OPA2Vec and Node2Vec) as well as the application to the prediction of PPIs and gene–disease associations, and the results may change with different application domains.

Nevertheless, our study allows us to draw several conclusions. First, our results demonstrate that including ontology axioms may add background knowledge that can significantly improve different prediction tasks. Consequently, our results can be used to improve the axioms as well as textual definitions and labels in existing ontologies. For example, we find that the axioms in ChEBI contribute significantly to the prediction of PPIs because ChEBI axioms reveal relationships between GO classes that are associated with the same chemical entities but that are not directly related in the GO hierarchy. The axioms also add information in a context-specific manner; for example, axioms from the PO only contribute to predicting protein interactions in *Arabidopsis* but no other taxa since PO contains plant-specific domain knowledge. Axioms may also add noise to a prediction if they are not well aligned with the prediction task. For example, axioms in the PATO ontology, despite PATO being significantly smaller in size than ChEBI, do not improve or even decrease performance across several applications.

We also find some evidence that there can be a performance difference when incorporating ontology meta-data into the data analysis. For example, when the OWL annotation axioms of ChEBI are included, the overall PPI prediction performance drops; the labels and definitions in ChEBI often consist of chemical formulas and other properties expressed in symbols or in a mathematical form (e.g. synonyms such as ‘(5Z,8Z,11Z,13E,15R)-15-hydroxyicosa-5,8,11,13-tetraenoic acid’ which are not well represented in literature and therefore not exploited well by our methods). One possibility to overcome this limitation would be combine our embedding method with a chemical named entity recognition and normalization method.

Including the meta-data (labels, definitions, synonyms, etc.) of the PATO ontology in the embeddings consistently decreases predictive performance across all our applications; a possible explanation for this observation is that the labels and definitions in PATO are not well aligned with any of the tasks we intend to perform; our approach provides a quantitative measure that can be used to improve the PATO definitions and labels for our tasks if this is deemed desirable by the PATO developers.

## 5 Conclusions

We evaluated the contribution of axioms in biomedical ontologies towards predictive analysis methods. In our experiments, we do not alter the biological data used for training and evaluation but only alter the background knowledge encoded in ontologies, using a set of data-driven methods that can encode entities with their ontology-based annotations, together with the ontologies and their axioms, within vector spaces. We find that the background knowledge contained in ontologies has the potential to significantly improve data analysis and machine learning in at least two distinct tasks in bioinformatics: exploiting functional similarity of proteins to predict PPIs, and exploiting phenotypic similarity between genotypes and diseases to predict gene–disease associations. While our analysis is limited to two tasks, many bioinformatics workflows utilize functional or phenotypic similarity over ontologies and may similarly benefit from exploiting the knowledge contained in richly axiomatized ontologies.

Our results have implications on the further development of knowledge bases and ontologies in the life sciences, in particular as machine learning methods are more frequently applied across the life sciences. Our findings motivate the need for further development, and the systematic, application-driven evaluation and improvement, of formal axioms in biomedical ontologies.

## Funding

The work was supported by the King Abdullah University of Science and Technology (KAUST) Office of Sponsored Research (OSR) [award numbers FCC/1/1976-04, FCC/1/1976-06, FCC/1/1976-17, FCC/1/1976-18, FCC/1/1976-23, FCC/1/1976-25, FCC/1/1976-26, URF/1/3450-01 and URF/1/3454-01].

*Conflict of Interest*: none declared. 

## Supplementary Material

btz920_Supplementary_Data
