# Supplementary material for: Formal axioms in biomedical ontologies improve analysis and interpretation of associated data
Source: Bioinformatics. 2019 Dec 10;36(7):2229–36. doi: 10.1093/bioinformatics/btz920 (PMC7141863; doi:10.1093/bioinformatics/btz920)
Supplement: btz920_Supplementary_Data [file btz920_supplementary_data.pdf]

Bioinformatics
doi.10.1093/bioinformatics/xxxxxx
Advance Access Publication Date: Day Month Year
Manuscript Category
OXFORD

Subject Section

Supplementary Materials: Formal axioms in biomedical ontologies improve analysis and interpretation of associated data

Fatima Zohra Smaili [1], Xin Gao [1,*] and Robert Hoehndorf [1,*]

[1] King Abdullah University of Science and Technology (KAUST), Computational Bioscience Research Center (CBRC), Computer, Electrical & Mathematical Sciences and Engineering (CEMSE) Division, Thuwal 23955, Saudi Arabia.

[*] To whom correspondence should be addressed.

Associate Editor: XXXXXXX

Received on XXXXX; revised on XXXXX; accepted on XXXXX

Abstract

1 Protein-protein interaction prediction using GO-plus

The AUC values for predicting PPIs based on the comprehensive set of axioms in GO, all axioms in each ontology and all axioms linking GO classes to each specific ontology classes are shown in Table 1.

2 Ontologies

We provide below a detailed description for each one of the biomedical ontologies linked to GO in GO-plus:

2.1 The ChEBI Ontology

We downloaded ChEBI in the OWL format from `http://purl.obolibrary.org/obo/chebi.owl` on April 26, 2018. The ChEBI ontology formally describes relations between molecular entities, in particular small chemical compounds (Degtyarenko *et al.*, 2007). It contains a total of 432,822 logical axioms and 92,015 classes.

2.2 The Plant Ontology (PO)

We downloaded the OWL version of PO from `http://purl.obolibrary.org/obo/po.owl` on April 26, 2018. This version of PO contains 4,835 axioms and 1,649 classes. PO provides a formal description of the vocabulary related to external and internal plant anatomy and plant development phases. It is mainly used to associate plant structures and development to gene expression and phenotype data (Cooper *et al.*, 2013).

2.3 The Cell Type Ontology (CL)

We downloaded CL in OWL from `http://purl.obolibrary.org/obo/cl.owl` on April 26, 2018. CL contains 17,958 axioms and 3,862 classes. It is an ontology that describes cell types for major animal and plant organisms (Bard *et al.*, 2005).

2.4 Phenotype and Trait Ontology (PATO)

The OWL version of PATO was downloaded from April 26, 2018 from `http://purl.obolibrary.org/obo/pato.owl`. This version contains 5,644 logical axioms and 2,251 different classes. PATO provides a systematic description of phenotypes through the concepts and relationships defined by its axioms (Gkoutos *et al.*, 2005).

2.5 Uberon Ontology

We downloaded the Uberon ontology on April 26, 2018 from `http://purl.obolibrary.org/obo/uberon.owl`. This OWL version of Uberon contains 65,067 logical axioms and 9,866 classes. Uberon is a multi-species anatomy ontology that describes anatomical structures across multiple species through manually-curated cross-references (Mungall *et al.*, 2012).

2.6 Sequence Ontology (SO)

We obtained the SO ontology from `http://purl.obolibrary.org/obo/so.owl` on November 25, 2018. This version of SO contains 5,443 logical axioms and 2,2234 classes. The SO consists of a set of classes and relations that describe the parts of a genomic annotation (Eilbeck *et al.*, 2005).

© The Author 2019.

	Human		Yeast		Arabidopsis	
	**Onto2Vec**	**Onto2Vec_NN**	**Onto2Vec**	**Onto2Vec_NN**	**Onto2Vec**	**Onto2Vec_NN**
GO (Baseline)	0.7660	0.8779	0.7701	0.8731	0.7559	0.8364
ChEBI	0.7905(+0.0245)	0.8911(+0.0132)	0.7920(+0.0219)	0.8854(+0.0123)	0.7721(+0.0162)	0.8534(+0.0170)
PO	0.7767(+0.0007)	0.8790(+0.0011)	0.7768(+0.0067)	0.8749(+0.0018)	0.7703(+0.0144)	0.8481(+0.0117)
CL	0.7804(+0.0144)	0.8793(+0.0014)	0.7823(+0.0122)	0.8758(+0.0027)	0.7619(+0.0060)	0.8374(+0.0010)
PATO	0.7781(+0.0121)	0.8788(+0.0009)	0.7711(+0.0010)	0.8738(+0.0007)	0.7569(+0.0010)	0.8402(+0.0038)
UBERON	0.7761(+0.0101)	0.8795(+0.0016)	0.7830(+0.0129)	0.8777(+0.0046)	0.7658(+0.0099)	0.8423(+0.0059)
SO	0.7890(+0.0230)	0.8788(+0.0009)	0.7768(+0.0067)	0.8793(+0.0062)	0.7612(+0.0053)	0.8391(+0.0027)
FAO	0.7703(+0.0043)	0.8781(+0.0002)	0.7712(+0.0011)	0.8738(+0.0007)	0.7560(+0.0001)	0.8373(+0.0009)
OBA	0.7657(-0.0003)	0.8821(+0.0042)	0.7874(+0.0173)	0.8804(+0.0073)	0.7567(+0.0008)	0.8379(+0.0015)
CARO	0.7742(+0.0032)	0.8829(+0.0050)	0.7890(+0.0189)	0.8809(+0.0078)	0.7631(+0.0072)	0.8511(+0.0147)
PR	0.7710(+0.0050)	0.8792(+0.0013)	0.7859(+0.0158)	0.8781(+0.0050)	0.7685(+0.0126)	0.8503(+0.0139)
NCBITaxon	0.7780(+0.0120)	0.8857(+0.0078)	0.7905(+0.0204)	0.8737(+0.0006)	0.7641(+0.0082)	0.8491(+0.0127)
Average Difference	(+0.0099)	(+0.0034)	(+0.0122)	(+0.0045)	(+0.0074)	(+0.0078)

Table 1. AUC values of the ROC curves for PPI prediction for each external ontology in GO-Plus using Onto2Vec and Onto2Vec-NN. Each prediction method uses all logical axioms from GO, all logical axioms from the referenced ontology, and all GO-Plus axioms describing relations between GO and the given ontology. The improvement (blue)/ decrease (red) in performance of each ontology compared to GO is shown between parentheses. The last row shows the average difference of the performance across all ontologies compared to the GO baseline.

2.7 Fungal Gross Anatomy Ontology (FAO)

We downloaded the FAO ontology on November 25, 2018 from `http://purl.obolibrary.org/obo/fao.owl`. The OWL version of FAO contains 155 axioms and 105 classes. The FAO describes the anatomy of fungi through a set of controlled vocabulary.

2.8 Ontology of Biological Attributes (OBA)

We downloaded the OBA ontology on November 25, 2018 from `http://purl.obolibrary.org/obo/oba.owl`. This ontology contains 73,377 axioms and 27,365 classes. OBA provides a collections of biological attributes.

2.9 NCBI organismal classification (NCBITaxon)

We obtained the NCBITaxon ontology from `http://purl.obolibrary.org/obo/ncbitaxon.owl`. This OWL version contains 3,653,676 axioms and 1,826,669 classes. This ontology provides a formal classification of different organisms (Federhen, 2011).

2.10 Commom Anatomy Reference Ontology (CARO)

The CARO ontology was obtained on `http://purl.obolibrary.org/obo/caro.owl` on November 25, 2018. This version contains 209 axioms and 158 classes. The CARO serves as a template to unify the structure of anatomy ontologies (Haendel *et al.*, 2008).

2.11 Protein Ontology (PR)

We downloaded the PR ontology from `http://purl.obolibrary.org/obo/pro_reasoned.owl` on November 4, 2018. This ontology contains 1,312,362 axioms and 400,923 classes. The PR ontology formally represents protein-related entities and their relations at different levels of specificity(Natale *et al.*, 2010).

Table 2 summarizes the number of axioms in GO-Plus describing relations to each of these ontologies and shows an example of such axioms for each ontology.

3 Node2vec

For comparison, we have applied node2vec (**?**) on the ontology graph and the class-entity relations to obtain embeddings for the biological entities used later in our analysis. Figure 3 shows the workflow used to apply Node2Vec in this work.

Figure 1 shows the detailed ROC curves when using node2vec to predict PPI based on GO and GO-plus for human, yeast and Arabidopsis datasets using cosine similarity and neural network. Figure 2 shows the ROC curves obtained for gene–disease association prediction using node2vec comparing Phenomenet (Phenomenet + GO) to Phenomenet combined with GO-plus (Phenomenet + GO-plus) for human and mouse datasets.

4 Evaluation metrics

We used the ROC (receiver operating characteristic) curve (Yin and Vogel, 2017) along with the AUC (area under ROC curve) as a quantitative measure to assess the performance of each predictive method. For both PPI prediction and gene–disease prediction, the true positive pairs are considered to be the ones available from the STRING network and the `MGI_DO.rpt` file from the MGI database respectively. The negative pairs on the other hand are down-sampled from the set of all unknown associations to form a set of negatives equal in size to the set of positive pairs for both PPI prediction and gene–disease association prediction.

5 Cosine similarity

One way to perform prediction tasks using ontology-based embeddings is by calculating the similarity between each pair of vectors and using the obtained similarity as a confidence score to predict whether two entities are associated or not. To do so, we use cosine similarity as a similarity measure between the obtained vectors. The cosine similarity cos_{sim} between two vectors A and B is calculated as

$$cos_{sim}(A, B) = \frac{A \cdot B}{\|A\| \, \|B\|}, \quad (1)$$

where $A \cdot B$ is the dot product of A and B.

Ontology	Number of axioms	Example
ChEBI	69,673	`’GDP-L-fucose biosynthetic process’ EquivalentTo ’biosynthetic process’ and (’has output’(some GDP-L-fucose ))`
PO	935	`’metaxylem development’ SubClassOf (’results in development of’ (some metaxylem ))`
CL	3,859	`’epithelial cell differentiation’ SubClassOf (’results in acquisition of features of’ (some ’epithelial cell’ ))`
PATO	205	`’supramolecular polymer’ SubClassOf (’bearer of’ (some polymeric))`
UBERON	17,132	`’mammary gland development’ SubClassOf (’results in development of’ ( some ’mammary gland’))`
SO	239	`’box C/D snoRNA metabolic process’ EquivalentTo (’metabolic process’ and has participant (some ’box C/D snoRNA’))`
FAO	99	`’cleistothecium development’ SubClassOf (results in development of some ’cleistothecium’)`
OBA	558	`’Regulation of post-lysosome vacuole size’ SubClassOf (regulates (some ’post-lysosomal vacuole size’))`
CARO	315	`’Anatomical structure development’ EquivalentTo (’Developmental process’ and ( results in development of ’anatomical structure’))`
PR	1,914	`’tyrosine 3-monooxygenase kinase activity’ SubClassOf (has input some (’tyrosine 3-monooxygenase’))`
NCBITaxon	1,136	`’chloroplast proton-transporting ATP synthase complex assembly’ SubClassOf (only_in_taxon Viridiplantae)`

Table 2. Number of inter-ontology axioms (with an example) in GO-Plus corresponding to each external ontology.

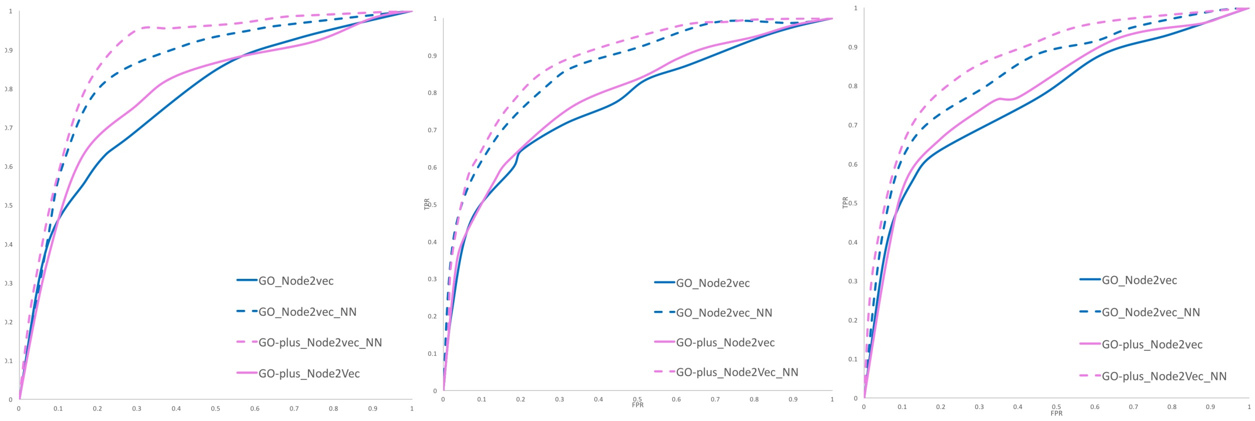

(a) Human (b) Yeast (c) Arabidopsis Thaliana

Fig. 1: ROC curves for PPI prediction using Node2vec for GO and GO-Plus for human, yeast, and Arabidopsis Thaliana using cosine similarity and the neural network.

Parameter	value
Dimension	200
min_count	1
window	10
walk_length	16
num_walks	100

Table 3. Parameter used for training the Node2Vec model.

References

Bard, J. *et al.* (2005). An ontology for cell types. *Genome biology*, **6**(2), R21.

Cooper, L. *et al.* (2013). The plant ontology as a tool for comparative plant anatomy and genomic analyses. *Plant and Cell Physiology*, **54**(2), e1.

Degtyarenko, K. *et al.* (2007). Chebi: a database and ontology for chemical entities of biological interest. *Nucleic acids research*, **36**(suppl_1), D344–D350.

Eilbeck, K. *et al.* (2005). The sequence ontology: a tool for the unification of genome annotations. *Genome biology*, **6**(5), R44.

Federhen, S. (2011). The ncbi taxonomy database. *Nucleic acids research*, **40**(D1), D136–D143.

Gkoutos, G. V. *et al.* (2005). Using ontologies to describe mouse phenotypes. *Genome biology*, **6**(1), R8.

Haendel, M. A. *et al.* (2008). Caro–the common anatomy reference ontology. In *Anatomy Ontologies for Bioinformatics*, pages 327–349. Springer.

Hunter, D. *et al.* (2012). Selection of proper neural network sizes and architectures—a comparative study. *IEEE Transactions on Industrial*

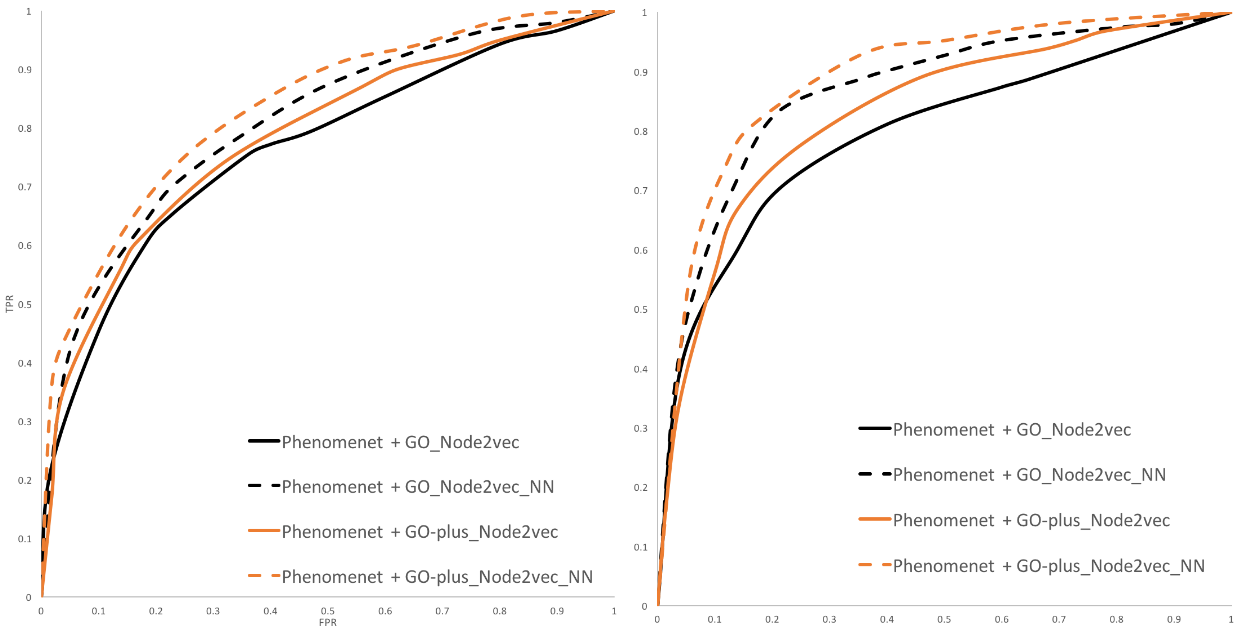

(a) Human (b) Mouse

Fig. 2: ROC curves for gene–disease prediction using node2vec comparing PhenomeNET with GO (PhenomeNET + GO) to PhenomeNET with GO-Plus (PhenomeNET + GO-plus) with cosine similarity (Cos) and with a neural network (NN) for human and mouse gene–disease associations.

Informatics, **8**(2), 228–240.

Mungall, C. J. *et al.* (2012). Uberon, an integrative multi-species anatomy ontology. *Genome biology*, **13**(1), R5.

Natale, D. A. *et al.* (2010). The protein ontology: a structured representation of protein forms and complexes. *Nucleic acids research*, **39**(suppl_1), D539–D545.

Yin, J. and Vogel, R. L. (2017). Using the roc curve to measure association and evaluate prediction accuracy for a binary outcome. *Biometrics & Biostatistics International Journal*, **5**(3), 1.

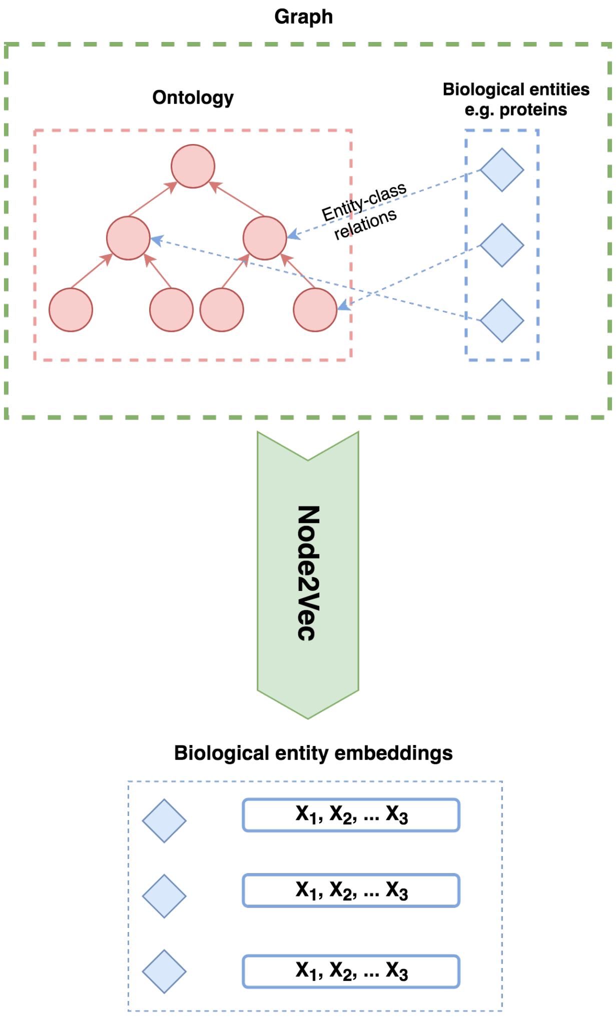

Fig. 3: Workflow for protein–protein interaction (PPI) prediction using OPA2Vec.
